# Ultrasound-guided “hourglass-pattern” fascia iliac block combined with sacral plexus and gluteal epithelial nerve block for an elderly hip fracture patient with organ failure

**DOI:** 10.1097/MD.0000000000019732

**Published:** 2020-06-19

**Authors:** Huiyue Wang, Qianyu Li, Yong Ni

**Affiliations:** a Department of Anesthesiology, Affiliated Hospital of Hebei University, Baoding, Hebei Province; b Department of Anesthesiology, The Second Affiliated Hospital of Suzhou University, Suzhou, Jiangsu Province, P.R. China.

**Keywords:** “hourglass-pattern” fascia iliac block, gluteal epithelial nerve block, hip fracture, internal fixation with proximal femoral intramedullary nail, sacral plexus block, ultrasound

## Abstract

**Introduction:**

Anesthesia management for high-risk elderly patients with hip fracture is challenging, it is significant to choose a more minimally invasive anesthesia technique for them than using conventional methods, like general anesthesia and neuraxial anesthesia.

**Patients concerns:**

Herein the patient suffered from the right intertrochanteric fracture, combined with heart failure, renal failure at the stage of uremia and pneumonia in her upper left lung

**Diagnosis:**

Because of right intertrochanteric fracture, internal fixation with proximal femoral intramedullary nail was scheduled for this patient

**Interventions:**

Ultrasound-guided “hourglass-pattern” fascia iliac block combined with sacral plexus and gluteal epithelial nerve block were performed to a high-risk elderly patient

**Outcomes:**

The surgery in our report was successfully completed with our effective anesthesia technique and no perioperative complication occurred

**Conclusion:**

Ultrasound-guided “hourglass-pattern” fascia iliac block combined with gluteal epithelial nerve block and sacral plexus block not only satisfied the anesthesia and provided effective postoperative analgesia of hip operation, but also has minimal invasion to high-risk elderly patients, and contributed to enhancing recovery after surgery

## 1 Introduction

The elderly patients with hip fracture often have multiple basic diseases, and some high-risk patients even have serious organ failure.^[1,2]^ Anesthesia methods affect the prognosis of patients,^[3]^ so the choice of anesthesia methods is very crucial. The best perioperative pain management for hip surgery can improve the long-term quality of life of patients.^[4]^ Compared with general anesthesia and neuraxial anesthesia, peripheral nerve block is minimally invasive, and can provide effective perioperative analgesia, which has its unique advantages.^[5]^

Some scholars reported^[6]^ that a combination of T12 paravertebral block and lumbar plexus, sacral plexus block can provide effective anesthesia and analgesia for hip surgery, but this anesthesia method requires to place the patient in the lateral position with surgical side uppermost, which will cause pain, discomfort and the possibility of complications in patients with hip fracture. Our report describes a case of internal fixation with proximal femoral intramedullary nail (PFN) for intertrochanteric fracture of femur in an elderly patient with heart failure and renal failure at the stage of uremia. We successfully performed ultrasound-guided “hourglass-pattern” fascia iliac block combined with sacral plexus and gluteal epithelial nerve block to this patient.

## 2 Case description

### 2.1 Patient consent for publication and ethical approval

Ethical approval (BD-2019083) for this report was provided by the Ethical Committee of No.1 Central Hospital of Baoding. Prior to the surgery, the patient had signed the written informed consent and approved us to public the details of this case.

### 2.2 Patient characteristics

A 73-year-old female (body height 148 cm, body weight 86 kg, BMI 39.3 kg/m^2^, American Society of Anesthesiologists status III) was scheduled for internal fixation with PFN for right intertrochanteric fracture. She had a history of diabetes mellitus for the past 20 years. She was at the stage of renal failure uremia for 3 years, and dialysis was carried out regularly 2 to 3 times a week at present. She also had a history of hypertension for 10 years, and oral nifedipine was used to control blood pressure. Prior to surgery, pneumonia in her upper left lung and heart failure were diagnosed, and she could not lie down. The BNP test results were 8655.00 pg/mL. Preoperative examination: HGB83.1 g/L, K5.62 mmol/L. GLU 11.60 mmol/L. Cardiac Doppler ultrasonography showed EF 45%, and moderate pulmonary artery hypertension. The VAS scores were 7 to 8 when she moved her injured limb slightly pre-operation.

### 2.3 Anesthetic technique

After the patient entered the operation room, venous access was established. Then a monitor of vital signs was connected to her. The patient^'^s baseline blood pressure was 168/95 mm Hg, heart rate was 83 beats/min, and oxygen saturation was 94%. Radial artery puncture was performed after local anesthesia infiltration to monitor invasive arterial blood pressure, and we measured her arterial blood gas analysis (Table 1). Dexmedetomidine was continuously pumped for 20 minutes at a rate of 0.6 to 0.8 ug/kg, then it was infused at the rate of 0.2 to 0.5 mg/kg/h. We used a face mask to administer supplemental oxygen at 3 L/min.

**Table 1:**
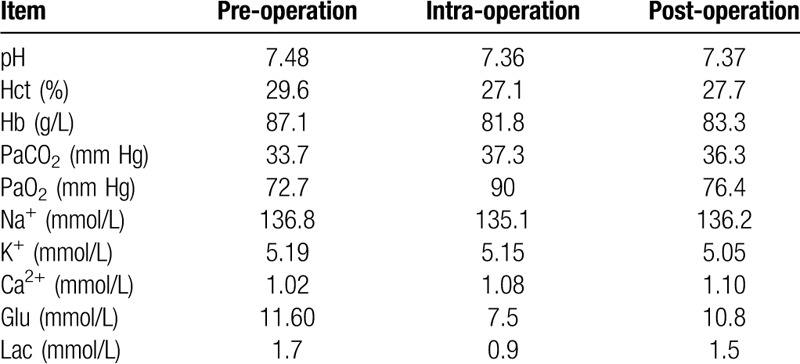
Arterial blood gas analysis.

### 2.4 Tab Arterial blood gas analysis

**Figure 1:**
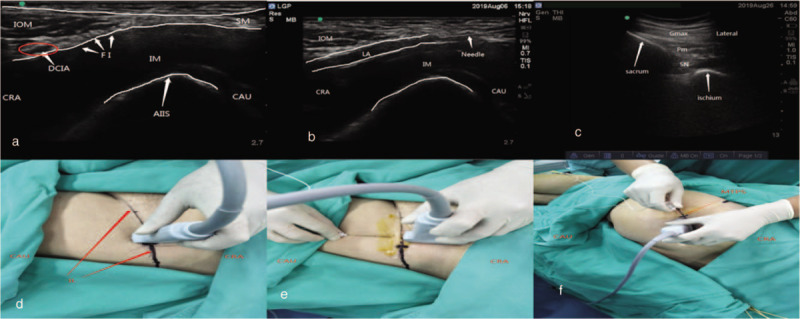
Ultrasonography, the positions of transducer and puncture point. **a:** ultrasonography of hourglass-pattern. **b:** ultrasonography of correct injection position of local anaesthetic during hydro-dissection technique. **c:** ultrasonography of sacral plexus, after injecting local anaesthetic. **d, e:** the positions of transducer and the puncture point of “hourglass-pattern” fascial iliac block **f:** the positions of transducer and the puncture point of sacral plexus block. AIIS = anterior inferior iliac spine, CAU = caudal, CRA = cranial, DCIA = deep circumflex iliac artery, FI = fascia iliaca, Gmax = gluteus maximus muscle, IM = iliopsoas muscle, IOM = internus obiquus abdominis muscle, LA = local anaesthetic, MTPS = midpoint of the line between greater trochanter of femur and the posterior superior iliac spine, PM = piriformis muscle, SM = sartorius muscle, SN = sacral plexus.

After 30 minutes of sedation, ultrasound-guided peripheral nerve block was performed. First, the patient was positioned in a supine position, and we performed “hourglass-pattern” fascial iliac block reported by Singh .^[7]^ A high-frequency ultrasound probe was vertically positioned in the middle and outer third of inguinal ligament, with the midpoint of the probe located above the inguinal ligament (Fig. 1d), identifying the important anatomical structures of internal oblique muscle of abdomen, sartorius muscle, and iliopsoas muscle, and found out the “hourglass-pattern” (Fig. 1a). After injecting local anesthesia at puncture point, we inserted a 9 cm 22G needle from the caudal to cranial, parallel to the probe, in-plane, and the needle advanced through the fascia iliac at the level of the inguinal ligament (Fig. 1e). With the technique of hydro-dissection and after confirming the injection location was correct, we injected 40 mL 0.33% ropivacaine, making sure that there was no intra-vascular injection. The correct injection position of local anesthetic should separate off fascia iliac from iliopsoas muscle, and the liquid should diffuse from caudal to cranial (Fig. 1b).

Five minutes later, through pinprick test, we confirmed that the block worked. Then the patient was placed in the lateral position with the surgical side uppermost, and the VAS scores were 0 to 1 during this process.

Sacral plexus block was performed with an easy approach as reported previously.^[8]^ In brief, the low-frequency ultrasound probe was placed in inner 1/2 of the line between the greater trochanter of femur and the posterior superior iliac spine. After moving the probe caudally we could find the greater sciatic foramen and the piriformis (Fig. 1c). In the deep of the piriformis, the sacral plexus was identified as a hyperechoic structure. The needle was inserted and advanced via in-plane technique. When the needle tip moved toward the deep of piriformis and reached the target nerve, we injected 20 mL 0.5% ropivacaine with negative aspiration throughout (Fig. 1f).

**Figure 2:**
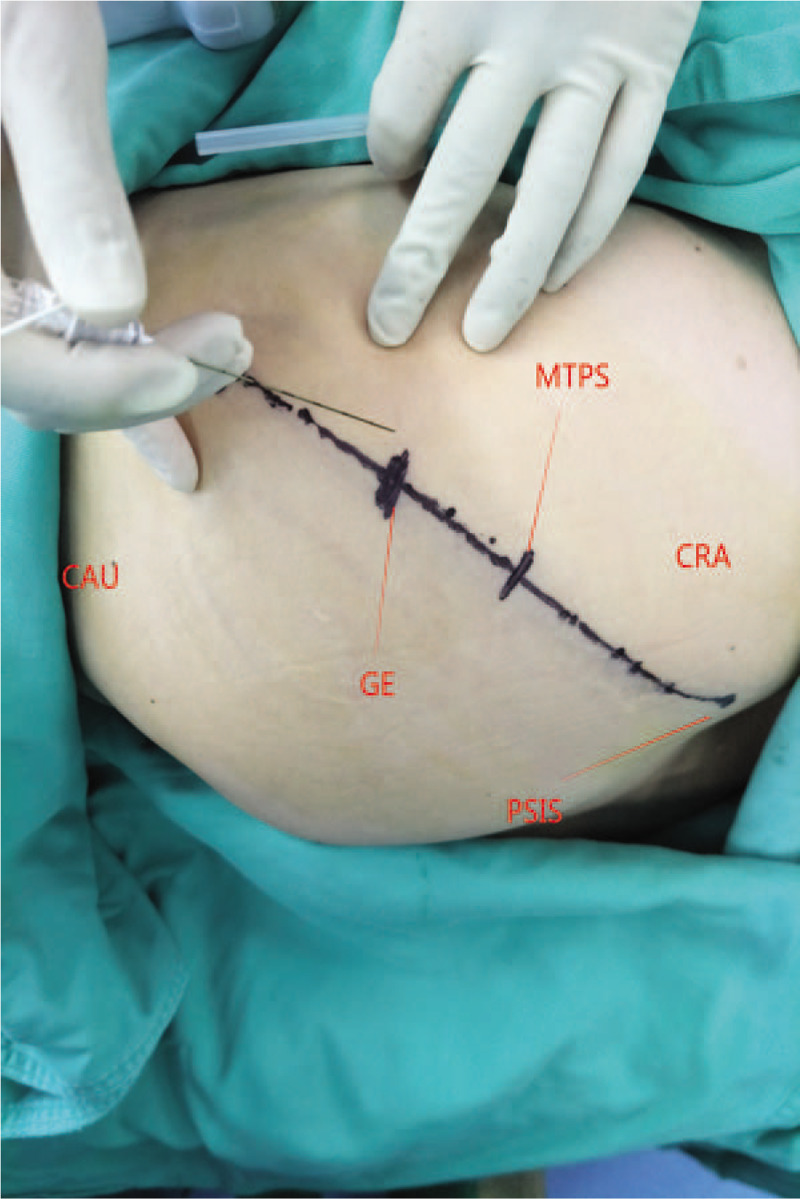
GE = the middle and outer third of the line between the posterior superior iliac spine and the greater trochanter of the femur (the superficial projection of the gluteal epithelial nerve), CAU = caudal, CRA = cranial, MTPS = midpoint of the line between greater trochanter of femur and the posterior superior iliac spine, PSIS = posterior inferior iliac spine.

As we know, the point at the middle and outer third of the line between the posterior superior iliac spine and the greater trochanter of the femur is the superficial projection of the gluteal epithelial nerve (Fig. 2). At this point, the gluteal epithelial nerve was blocked by local infiltration of 5 mL 1% lidocaine.

About 18 minutes later, all the nerve block we performed were completely effective by pinprick test. During the operation, dexmedetomidine was pumped at the speed of 0.2 to 0.5 mg/kg/h, and arterial blood gas analysis was measured (Table 1). The duration of surgery was 68 minutes. No other analgesics were used during the operation.

### 2.5 Post operation

In postanesthesia care unit , no additional analgesia was used. Arterial blood gas analysis was measured at night after the patient returned to the ward (Table 1). The duration of nerve block was 10 hours, after which postoperative analgesia was performed in multiple modes, and the VAS scores were 0 to 1. Day 2 after operation, the BNP test results were 5708.00 pg/mL. Postoperative examination: HGB81.8 g/L, K4.95 mmol/L, and the symptoms of pneumonia in her left lung did not get worse. This patient discharged on the fifth day after surgery. No postoperative complication occurred.

## 3 Discussion

The elderly patients with hip fracture frequently have multiple comorbidities, therefore, although early surgery is performed on them, the postoperative mortality and disability rates are still high.^[9]^ Karaca et al^[10]^ have shown that anesthesia method is an independent factor affecting the mortality of hip surgery. The principle for anesthesia selection is to reduce or avoid the effect of anesthesia on systemic and vital organ functions as much as possible, in the condition that the needs of surgery are met. As routine anesthesia methods, general anesthesia and neuraxial anesthesia have more limitations, contraindication and complications.^[11]^ By contrast with them, peripheral nerve block has little influence on hemodynamics and causes less complication, especially for some high-risk patients with cardiopulmonary dysfunction.^[12]^

Ueshima^[13]^ reported that supra-inguinal fascia iliac block under ultrasound guidance can provide effective perioperative analgesia for hip surgery. For the incision of internal fixation with PFN, blockade of the lateral femoral cutaneous nerve is crucial. And some studies have shown that “hourglass-pattern” fascial iliac block can effectively diffuse to the cranial and block the lateral femoral cutaneous nerve and femoral nerve 100%.^[14]^ However, the internal fixation with PFN for intertrochanteric fracture was closed reset under C-arm X-ray fluoroscopy first. After a satisfactory reset, a 5 cm longitudinal incision was made at the place 5 to 10 cm above the greater trochanter, where the superficial projection of the gluteal epithelial nerve is almost located. Through this incision, the gluteus medius and gluteus minimus innervated by sacral plexus were separated, the apex of the greater trochanter of femur was exposed, the greater trochanter of femur was opened and an intramedullary nail was inserted into it, then the intramedullary nail was locked at the distal end.^[15]^ Because of these incisions, the anesthetic technique, fascia iliac block combined with gluteal epithelial nerve and sacral plexus block, is needed.

In this case, the patient, an elderly and critically ill one, has heart failure, renal failure at uremia stage, and pneumonia in her upper left lung. She also has a history of diabetes mellitus and hypertension for many years, any stress response may cause serious or even fatal cardiovascular accidents. Therefore, perioperative management of anesthesia for her is extremely difficult. The method of injecting dexmedetomidine for mild sedation, not only helps to alleviate anxiety of patients, but also has positive effect on recovery of elderly patients.^[16]^ Then we performed ultrasound-guided “hourglass-pattern” fascial iliac block, as described in the method. Compared with ultrasound-guided lumbar combined with sacral plexus block, which has been widely used in hip fracture surgeries, this method is easy to operate and also has advantage in image recognition. The position of fascial iliac is superficial and there is no need to change position when we perform this procedure. More importantly, via this block, ropivacaine can effectively diffuse from caudal to cranial, and the lateral femoral cutaneous nerve and the femoral nerve can be blocked 100%.^[7,14]^ Ultrasound-guided “hourglass-pattern” fascial iliac block can block the main branches of the lumbar plexus, but it causes less complications and is easier in ultrasound localization than posterior lumbar plexus block.^[17–18]^ Performing “hourglass-pattern” fascial iliac block successfully, can significantly improve the hip pain in the patient. It also provides analgesia for placing the patient in lateral supine position to block sacral plexus and gluteal epithelial nerve, and may avoid the hemodynamic changes caused by this process. The vital signs of the patient are more stable in this way. During the operation, dexmedetomidine was still used for continuous sedation. No discomfort was caused, the hemodynamics was stable and no ephedrine vasopressor was used. Gluteal epithelial nerve block, sacral plexus block combined with “hourglass-pattern” fascia iliac block not only satisfied the anesthesia of operation, but also provided effective postoperative analgesia, and did not affect patient's early ankle movement,^[14,19]^ which contributed to enhancing recovery after surgery.

Although this anesthesia method is successful in perioperative management of this high-risk elderly patient with hip fracture, there are still some shortcomings. Firstly, there is no prospective randomized controlled study, which is needed to provide sufficient evidence. Secondly, the effective and safe concentration and volume of sacral plexus block and “hourglass-pattern” fascial iliac block are still uncertain. Thirdly, we 555 did not perform gluteal epithelial nerve block with the guiding of ultrasound; we should try to utilize ultrasound to block this nerve later. In addition, it is very important to locate the needle tip during the puncture process. We are collecting more samples presently, and hope that in the future, sufficient evidence will be provided via more prospective clinical trials study.

## Acknowledgments

The authors thank the Second Affiliated Hospital of Suzhou Medical University for cooperation.

## Author contributions

**Conceptualization:** Qianyu Li, Yong Ni.

**Formal analysis:** Huiyue Wang.

**Investigation:** Huiyue Wang.

**Writing – original draft:** Huiyue Wang.

**Writing – review & editing:** Huiyue Wang, Qianyu Li, Yong Ni.
